# Externalising pathways to alcohol‐related problems in emerging adulthood

**DOI:** 10.1111/jcpp.13167

**Published:** 2019-11-25

**Authors:** Gemma Hammerton, Alexis C. Edwards, Liam Mahedy, Joseph Murray, Barbara Maughan, Kenneth S. Kendler, Matthew Hickman, Jon Heron

**Affiliations:** ^1^ Population Health Sciences Bristol Medical School University of Bristol Bristol UK; ^2^ Virginia Institute for Psychiatric and Behavioral Genetics Virginia Commonwealth University Richmond VA USA; ^3^ Postgraduate Program in Epidemiology Federal University of Pelotas Pelotas Rio Grande do Sul Brazil; ^4^ MRC Social, Genetic & Developmental Psychiatry Centre King's College London London UK

**Keywords:** Avon Longitudinal Study of Parents and Children, alcohol‐related problems, conduct problems, criminal behaviour, mediation

## Abstract

**Background:**

Both ‘early‐onset persistent’ and ‘adolescent‐onset’ conduct problems (CPs) are associated with alcohol‐related problems in emerging adulthood. The escalation of early CPs into criminal behaviour and heavy alcohol consumption prior to emerging adulthood are both likely to be important pathways.

**Methods:**

Data were analysed from 3,038 young people in a UK birth cohort, the Avon Longitudinal Study of Parents and Children. The exposure was developmental trajectories of CPs (‘low’, ‘childhood‐limited’, ‘adolescent‐onset’ and ‘early‐onset persistent’) between ages 4 and 13 years. The mediator was latent classes representing heavy alcohol consumption and/ or criminal behaviour at age 15 years. For the outcome, a quadratic latent growth curve was estimated to capture nonlinear change in alcohol‐related problems between ages 18 and 23 years.

**Results:**

Those with ‘early‐onset persistent’ [*b*(95% CI) = 1.16 (0.17, 2.14)] and ‘adolescent‐onset’ CPs [*b*(95% CI) = 1.31 (0.17, 2.45)] had higher levels of alcohol‐related problems at age 18 years compared to those with ‘low’ CPs’, but there was little evidence of an association with alcohol‐related problems after age 19 years. There was evidence for an indirect effect of ‘early‐onset persistent’ CPs [*b*(95% CI) = 1.12 (0.52, 1.72)] on alcohol‐related problems at age 18 years via the latent classes of alcohol and criminal behaviour in adolescence. This was not found for ‘adolescent‐onset’ CPs [*b*(95% CI) = 0.35 (−0.36, 1.07)].

**Conclusions:**

Strong associations exist between early CPs, adolescent alcohol consumption and criminal behaviour and alcohol‐related problems at age 18 years. Associations between early CPs and alcohol‐related problems weakened considerably across emerging adulthood.

## Introduction

Alcohol‐related problems peak in the early 20s (Brown et al., 2008; Derefinko et al., 2016; Maggs & Schulenberg, 2004) and excessive drinking at this age is associated with a wide range of short‐ and long‐term harms to health, including injury, risky sexual behaviour, mental health disorders and increased likelihood of alcohol abuse and dependence later in life (Ellickson, Tucker, & Klein, 2003; McCambridge, McAlaney, & Rowe, 2011). Reducing harmful levels of drinking in early adulthood, and its adverse consequences, is challenging; therefore, identifying risk factors earlier in life is essential for successful prevention strategies.

Conduct problems (CPs) are one of the strongest and most robust childhood predictors of both the initiation of regular alcohol use in adolescence (Dodge et al., 2009; Englund, Egeland, Oliva, & Collins, 2008) and problematic use in adulthood (Edwards, Gardner, Hickman, & Kendler, 2015; Englund et al., 2008; Zucker, 2008). The importance of the developmental timing of CPs is well established with a highly influential taxonomy developed by Moffitt (Moffitt, 1993; Moffitt, Caspi, Harrington, & Milne, 2002) distinguishing between those whose CPs begin in adolescence (‘adolescent‐onset’), those whose CPs manifest throughout childhood and adolescence (‘early‐onset persistent’), and those with little or no CPs (‘low’). The theory states that those with ‘early‐onset persistent’ CPs will show the worst long‐term outcomes (including problematic alcohol use), whereas the ‘adolescent‐onset’ group will mature out of their conduct (and related problems) during the transition to adulthood, providing certain snares, such as substance use or a criminal record, are not encountered (Moffitt, 1993). A recent metaanalysis found that both those with ‘early‐onset persistent’ and ‘adolescent‐onset’ CPs have a twofold increased odds of drinking excessive amounts of alcohol in early adulthood, compared to those with ‘low’ CPs (Bevilacqua, Hale, Barker, & Viner, 2017). One explanation for these findings is that turning points (such as starting a career, getting married and having children) marking the transition to adulthood are occurring later (during a development period from age 18 to 25 years known as emerging adulthood; Arnett, 2000), delaying desistence from problem behaviour for the ‘adolescent‐onset’ group until the late‐twenties (Moffitt et al., 2002; Reckdenwald, Ford, & Murray, 2016).

Although the associations are well established, less is known about the processes linking childhood CPs and later alcohol‐related problems. Both heavy alcohol use and gaining a criminal record are thought to act as potential ‘snares’, trapping those with early CPs into experiencing persisting problems into adulthood (Moffitt, 1993). Therefore, the escalation of early CPs into criminal behaviour in late adolescence, and heavy alcohol consumption, especially when occurring in combination with criminal behaviour, are both likely to be important mechanisms (Buchmann et al., 2009; Englund et al., 2008; Pitkänen, Lyyra, & Pulkkinen, 2005; White, 2015).

Examining whether co‐occurring alcohol use and crime in adolescence mediate the association between childhood CPs and alcohol‐related problems in emerging adulthood will inform prevention strategies for alcohol‐related problems. Although there is some evidence that childhood CPs can be treated or prevented (for example, using early prevention strategies focusing on improving social and emotional development in the child and targeting parent factors; Henggeler, 2015; Schindler & Black, 2015), targeting the short‐term consequences of CPs is also important to prevent ongoing problems into adulthood. These short‐term consequences often co‐occur in adolescence (Eklund & Klinteberg, 2009; White, 2015), and the importance of distinguishing the effects of heavy alcohol use on later negative outcomes from those of co‐occurring problem behaviours, such as criminal behaviour, has been highlighted (Brown et al., 2008). This will have implications for the best way to target these problem behaviours in adolescence. There is increasing support for prevention strategies in adolescence that target the consequences of CPs such as substance use (Kim, Gilman, & Hawkins, 2015), with the strongest evidence base for multicomponent prevention programmes [particularly universal school‐based interventions (Macarthur et al., 2018)] that seek to address factors in multiple domains such as substance use, aggression and violence in the classroom (Botvin, Griffin, & Nichols, 2006; Brown et al., 2008; Hahn et al., 2007; Le Blanc, 2015) and interventions that account for adolescents’ sensitivity to feeling high status and respect (Yeager, Dahl, & Dweck, 2018).

Identifying potential mediators of the association between childhood CPs and later alcohol‐related problems will also make an important contribution to the theory and wider literature on the developmental timing of childhood CPs. Little is known about whether the most important mechanisms (and potential intervention targets) are the same for those with ‘early‐onset persistent’ and those with ‘adolescent‐onset’ CPs. Previous literature suggests that ‘early‐onset persistent’ CPs show the strongest association with later criminal behaviour, followed by ‘adolescent‐onset’ CPs (Bor, McGee, Hayatbakhsh, Dean, & Najman, 2010; Moffitt, 1993; Odgers et al., 2008). There is also evidence that both criminal behaviour and alcohol use in adolescence may lead to the development of later alcohol‐related problems (Buchmann et al., 2009; Englund et al., 2008; Grant & Dawson, 1997; Hingson, Heeren, & Winter, 2006; Pitkänen et al., 2005; White, 2015) with community studies showing that early criminal behaviour is a stronger predictor of later alcohol problems than vice versa (White, 2015). One mechanism that could explain this is associating with a deviant peer group (Fergusson, Swain‐Campbell, & Horwood, 2002; Mahedy et al., 2018). Although heavy alcohol use in adolescence could be an earlier version of later alcohol problems, we viewed it as an important precursor to the development of alcohol problems (supported by a rich literature for example Buchmann et al., 2009; Englund et al., 2008; Grant & Dawson, 1997; Hingson et al., 2006; Pitkänen et al., 2005).

The present study investigates associations between developmental trajectories of childhood CPs, criminal behaviour and alcohol use in a prospective, population‐based birth cohort, the Avon Longitudinal Study of Parents and Children (ALSPAC). Previous studies using the ALSPAC sample have found that ‘early‐onset persistent’ and ‘adolescent‐onset’ CPs are associated with frequent alcohol use between 13 and 15 years (Heron et al., 2013) and broader substance use at age 18 years (Kretschmer et al., 2014) compared with ‘low’ CPs. We aim to further contribute to the literature in two ways. First, although the association between developmental trajectories of CPs and later alcohol‐related problems at a single point in time is well established (Bor et al., 2010; Kretschmer et al., 2014; Moffitt et al., 2002; Odgers et al., 2008), we are aware of no studies that have examined the association between trajectories of childhood CPs and the *longitudinal cours*e of alcohol‐related problems across emerging adulthood. By examining alcohol‐related problems longitudinally, we can investigate whether associations persist or weaken across this developmental period. Second, the mechanisms underlying the strong associations between developmental trajectories of CPs and later alcohol‐related problems are still unknown (Lahey, 2015; Wertz et al., 2018), and the importance of heavy alcohol use and criminal behaviour in adolescence (in isolation or when they co‐occur) has rarely been examined. We hypothesise that both ‘early‐onset persistent’ and ‘adolescent‐onset’ CPs will be associated with elevated alcohol‐related problems across emerging adulthood (age 18 to 23 years) and that these associations will be mediated, in part, by a combination of heavy alcohol consumption and criminal behaviour in adolescence (age 15 years).

## Methods

### Sample

Data were utilised from a large UK birth cohort, the ‘Avon Longitudinal Study of Parents and Children’ (ALSPAC) which was set up to examine genetic and environmental determinants of health and development (Boyd et al., 2013). ALSPAC recruited pregnant women resident in Avon, UK with expected dates of delivery 1 April 1991 to 31 December 1992. Of these initial 14,541 pregnancies, there was a total of 14,676 foetuses, resulting in 14,062 live births and 13,988 children who were alive at 1 year of age. The sample was restricted to singletons or first‐born twins, leaving a starting sample of 13,793. Parents and children have been followed up regularly since recruitment via questionnaire and clinic assessments. Study data from 2014 onwards were collected and managed using REDCap electronic data capture tools hosted at University of Bristol (Harris et al., 2009). Further details on the sample characteristics and methodology have been described previously (Boyd et al., 2013; Fraser et al., 2013), and detailed information about ALSPAC can be found on the study website (http://www.bristol.ac.uk/alspac). For information on all available ALSPAC data see the fully searchable data dictionary (http://www.bris.ac.uk/alspac/researchers/data-access/data-dictionary).

### Ethical considerations

Written, informed consent was obtained from all mothers who entered the ALSPAC study and ethical approval for the study was obtained from the ALSPAC Ethics and Law Committee (IRB00003312) and the Local Research Ethics Committees. The ethics committee specifically approved the questionnaires and the clinic testing protocols including the methods of gaining consent.

### Measures

**Figure 1:**
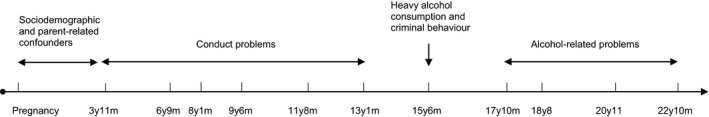
Timeline for data collection

Data were collected both during focus clinics and via questionnaires that were either returned by post or completed online. A timeline for data collection is shown in Figure 1.

#### Exposure: conduct problems across childhood

Developmental trajectories of CPs between ages four and 13 years have been derived previously (Barker & Maughan, 2009). Briefly, latent class growth analysis models were applied to six binary indicators of CPs derived from the ‘conduct problem’ scale of the Strengths and Difficulties Questionnaire (Goodman, 2001), which was dichotomised at the threshold of 4 or more. The items used to assess CPs at each time point included ‘often has temper tantrums or hot tempers’, ‘generally obedient, usually does what adults request’, ‘often fights with other children or bullies them’, ‘often lies or cheats’ and ‘steals from home, school or elsewhere’. The four resulting trajectories were described as ‘low’ (*n* = 4,737; 64%), ‘childhood‐limited’ (*n* = 1,060; 15%), ‘adolescent‐onset’ (*n* = 852; 12%) and ‘early‐onset persistent’ (*n* = 661; 9%).

#### Potential mediator: heavy alcohol consumption and criminal behaviour in adolescence

A self‐report battery of questions regarding criminal acts committed in the past year (Smith & McVie, 2003) was completed by the young person during a computer‐based session at a focus clinic at age ~ 15 years (mean = 15 years 6 months, standard deviation (*SD*) = 4 months). Construct validity for this self‐report questionnaire has been examined previously in adolescents using cross‐checks with agency records and teachers' questionnaires (Smith et al., 2001). Two items (carried a weapon and assault) were combined to create a binary measure of ‘violent crime’, and five items (stole from shops, broke into a vehicle or building, damaged property, sold illegal drugs and sold stolen goods) were combined to create a binary measure of ‘non‐violent crime’.

During the same computer‐based session, respondents reported detailed information about their alcohol consumption. The following binary variables for alcohol use were used, as defined previously in this sample (Melotti et al., 2013): ‘heavy typical drinking’ (using the question ‘in the last six months when you have had a drink, on a typical day how many drinks do you usually have’ with a cut point of more than four drinks per occasion), ‘frequent drinking’ (using the question ‘how many times have you had a full drink of alcohol in the last six months’ with a cut point of more than 20 times) and ‘regular binge drinking’ (using the question ‘in the last two years how many times have you had five or more drinks in 24 hours’ with a cut point of more than 20 occasions).

#### Outcome: alcohol‐related problems across emerging adulthood

The self‐report 10‐item Alcohol Use Disorders Identification Test (AUDIT (Babor, Higgins‐Biddle, Saunders, & Monteiro, 2001)), which is a brief screening tool to identify individuals with alcohol‐related problems, was completed by the young person at four time points between ages 18 and 23 years. At age ~ 18 years (mean = 17 years 10 months, *SD* = 5 months), data were collected during a computer‐based session at a focus clinic, and at ages ~ 19 years (mean = 18 years 8 months, *SD* = 6 months), ~21 years (mean = 20 years 11 months, *SD* = 6 months) and ~23 years (mean = 22 years 10 months, *SD* = 6 months), data were collected via online or postal questionnaire. The AUDIT scale has been studied extensively and has high validity and reliability in the detection of risky drinking, alcohol misuse and alcohol dependence (Allen, Litten, Fertig, & Babor, 1997). The AUDIT was treated as a continuous scale in analyses.

#### Potential confounders

Detail on the assessment of confounding factors (including child sex, maternal education, parity and adverse childhood experiences) is provided in Appendix S1.

### Statistical analysis

**Figure 2:**
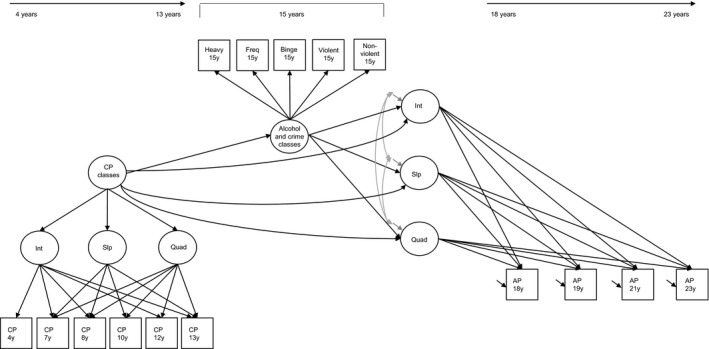
Structural equation model showing associations between latent classes of conduct problems (CPs) across childhood, latent classes of alcohol consumption and criminal behaviour in adolescence, and growth factors for alcohol‐related problems (APs) in emerging adulthood; CP: conduct problems; Heavy: heavy typical drinking; Freq: frequent drinking; Binge: regular binge drinking; Violent: violent crime; Nonviolent: nonviolent crime; AP: alcohol‐related problems; Int: intercept; Slp: linear slope; and Quad: quadratic; circles represent latent variable, and squares represent observed variable

The overall goal of this study was to examine the association between developmental trajectories of childhood CPs and alcohol‐related problems across emerging adulthood, and the extent to which these associations may be mediated through heavy alcohol consumption and criminal behaviour in adolescence. The final model is shown in Figure 2.

#### Potential mediator: latent class analysis for alcohol consumption and criminal behaviour in adolescence

Latent class analysis (LCA) was used to identify different patterns of alcohol consumption and criminal behaviour at age 15 years, using as class indicators, two binary measures of criminal behaviour (‘violent crime’ and ‘nonviolent crime’) and three binary measures of heavy alcohol use (‘heavy typical drinking’, ‘frequent drinking’ and ‘regular binge drinking’). Further detail on the LCA is given in Appendix S2.

#### Outcome: latent growth curve for alcohol‐related problems across emerging adulthood

Given that alcohol‐related problems increase and then decrease across emerging adulthood, a quadratic latent growth curve was estimated to capture nonlinear change from age 18 to 23 years. Time was specified as the number of years since age 18 and assumed to be the same for all individuals. Given that measurement error and day‐to‐day fluctuations in alcohol‐related problems are likely to remain constant across emerging adulthood, residual variances were constrained to be longitudinally invariant.

#### Estimating the association between conduct problem latent classes (X), latent classes of alcohol consumption and criminal behaviour (M), and alcohol‐related problems across emerging adulthood (Y)

The associations between the latent classes and the continuous distal outcome were estimated using the modified Bolck, Croon and Hagenaars (BCH) three‐step approach (Vermunt, 2010). This method uses a weighted multiple group analysis, where the groups correspond to the latent classes and the weights reflect the measurement error of the latent class variable. Therefore, this method accounts for the uncertainty in latent class assignment but prevents the shift in class distributions that is often seen using alternative approaches when the distribution of the continuous distal outcome is non‐Gaussian (Asparouhov & Muthén, 2014).

#### Estimating the indirect effect of conduct problem latent classes (X) on alcohol‐related problems across emerging adulthood (Y) via the latent classes of alcohol consumption and criminal behaviour (M)

The counterfactual approach to mediation was used to examine the extent to which the latent classes of alcohol consumption and criminal behaviour explained the association between childhood CPs and alcohol‐related problems in emerging adulthood. The counterfactual approach was necessary to enable indirect effects to be estimated via a nominal (e.g. latent class) mediator (Muthén, 2011). Further detail is provided in Appendix S3. All models were analysed in M*plus* v8 using robust maximum likelihood estimation (Muthén & Muthén, 2017). An annotated M*plus* script for the final analysis model is available on request.

#### Missing data

For the estimation of the latent growth curve for alcohol‐related problems, missing data were handled using full information maximum likelihood (FIML) estimation (Enders, 2010). Any respondent with at least one repeated measure of alcohol‐related problems (*N* = 6,178) was included in the latent growth curve analysis under the missing at random assumption (i.e. there are no systematic differences between observed and missing values for any dependent variable when conditioning on the remaining variables in the model)*.* The analysis sample consisted of those with data available for the exposure, mediator and confounding factors (*N* = 3,038; 1,348 males and 1,690 females). A flow chart of retention is shown in Appendix S4. Analyses were performed using inverse probability weighting (IPW) to address any potential bias caused by participant dropout (Seaman & White, 2013). Further information on the IPW analyses is given in Appendix S5. As it is not possible to incorporate bootstrapped confidence intervals and IPW into the same model, unweighted mediation analyses were also performed using bootstrapping.

## Results

Descriptive information for all observed variables used in analyses is shown in Appendix S6.

### Latent growth curve for alcohol‐related problems across emerging adulthood

Estimated and observed means for the alcohol‐related problems latent growth curve and means, variances and correlations between growth factors are given in Appendix S7. The alcohol‐related problems growth curve started at age 18 years with an average of 7.2 points (standard error (*SE*) = 0.07) on the AUDIT scale, and initially increased 1.4 units per year (*SE* = 0.06), before decreasing with an average quadratic factor of −0.3 (*SE* = 0.01). Associations between potential confounders and alcohol growth factors are shown in Appendix S1.

### Latent class analysis for alcohol consumption and criminal behaviour in adolescence

**Figure 3:**
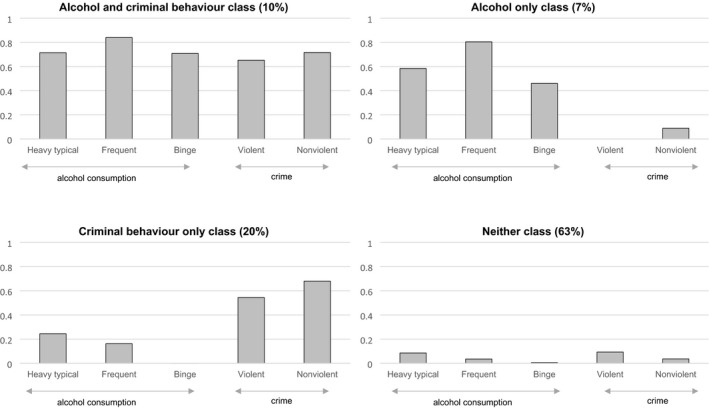
Latent classes of heavy alcohol consumption and criminal behaviour at age 15 years, and their constituent behaviours; *N* = 5,133

One‐ to five‐class models were compared for the latent classes of alcohol consumption and criminal behaviour at age 15 years. The four‐class model provided the best fit to the data (see Appendix S2 for model fit statistics and validation of the classes). Figure 3 shows the within‐class probabilities for each item (heavy typical consumption, frequent drinking, regular binge drinking, violent crime and nonviolent crime).

Ten per cent (*n* = 514) of the sample were classified as having a high probability of endorsing all items, 7% (*n* = 339) were classified as having a high probability of endorsing items related to alcohol consumption but not criminal behaviour, 20% (*n* = 1,049) were classified as having a high probability of endorsing items related to criminal behaviour but not alcohol consumption, and 63% (*n* = 3,232) were classified as having a low probability of endorsing any item.

#### Associations between conduct problem latent classes (X), latent classes of alcohol consumption and criminal behaviour (M), and alcohol‐related problems across emerging adulthood (Y)

**Figure 4:**
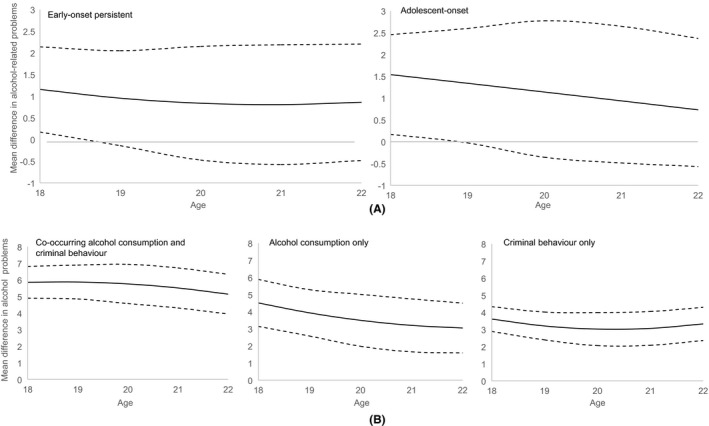
(A) Association between each conduct problem (CP) class and alcohol‐related problems from age 18 to 22 years; left figure shows ‘early‐onset persistent’ vs. ‘low’ CPs, right figure shows ‘adolescent‐onset’ vs. ‘low’ CPs; solid line shows mean difference in alcohol‐related problems across each class comparison, and dashed lines show 95% confidence intervals around the effect estimates. (B) Association between each class of age 15 alcohol and criminal behaviour and alcohol‐related problems from age 18 to 22 years; left figure shows ‘co‐occurring alcohol and criminal behaviour’ vs. ‘neither’, middle figure shows ‘alcohol consumption only’ vs. ‘neither’, right figure shows ‘criminal behaviour only vs. neither’; solid line shows mean difference in alcohol‐related problems across each class comparison and dashed lines show 95% confidence intervals around the effect estimates

There was strong evidence for an association between childhood CPs and initial levels of alcohol‐related problems at age 18 years (Wald χ^2^ (3) = 12.13; *p* = .007). Specifically, those with ‘early‐onset persistent’ [mean alcohol‐related problems: 8.24; *b*(95% CI) = 1.16 (0.17, 2.14)] and those with ‘adolescent‐onset’ CPs [mean alcohol‐related problems: 8.40; *b*(95% CI) = 1.31 (0.17, 2.45)] had higher levels of alcohol‐related problems at age 18 years compared to those with ‘low’ CPs [mean alcohol‐related problems: 7.09]. There was no association between ‘childhood‐limited’ CPs and alcohol‐related problems at age 18 years [mean alcohol‐related problems: 7.09; *b*(95% CI) = 0.01 (−0.85, 0.86)]. Figure 4A shows that the associations between childhood CPs and alcohol‐related problems (mean differences in alcohol problems for ‘early‐onset persistent’ vs. ‘low’ and ‘adolescent‐onset’ vs. ‘low’) weaken with age (from age 18 to age 22 years).

There was strong evidence for an association between childhood CPs and classes of alcohol consumption and criminal behaviour at age 15 (Wald χ^2^ (9) = 28.91; *p* < .001). Specifically, those with ‘early‐onset persistent’ CPs compared to those with ‘low’ CPs had increased odds of reporting both heavy alcohol consumption and criminal behaviour at age 15 years [OR (95% CI) = 3.37 (1.71, 6.67)], and of criminal behaviour without heavy alcohol consumption [OR (95% CI) = 3.70 (1.98, 6.92)] compared to reporting neither. All associations are shown in Table 1, and overlap between the two sets of latent classes is shown in Appendix S8.

**Table 1:** Association between childhood conduct problems (CPs) and latent classes of heavy alcohol consumption and criminal behaviour in adolescence showing multinomial odds ratio (95% confidence interval); *N* = 3,038

Latent classes Omnibus p < .001	Neither	Criminal behaviour only	Heavy alcohol only	Heavy alcohol and criminal behaviour
Low CPs	Reference	Reference	Reference	Reference
Childhood‐limited CPs	Reference	1.03 (0.46, 2.29)	0.87 (0.23, 3.30)	1.43 (0.66, 3.07)
Adolescent‐onset CPs	Reference	1.93 (0.84, 4.43)	0.80 (0.14, 4.73)	1.36 (0.44, 4.20)
Early‐onset persistent CPs	Reference	3.70 (1.98, 6.92)	0.99 (0.23, 4.31)	3.37 (1.71, 6.67)

Finally, there was an association between classes of alcohol consumption and criminal behaviour at age 15 years and initial levels of alcohol‐related problems at age 18 years (Wald χ^2^ (3) = 298.04; *p* < .001). Specifically, those that reported heavy alcohol consumption, criminal behaviour or both had higher levels of alcohol‐related problems at age 18 years compared with those that reported neither [both alcohol and crime: *b*(95% CI) = 5.86 (4.90, 6.81); alcohol only: *b*(95% CI) = 4.51 (3.14, 5.88); and crime only: *b*(95% CI) = 3.61 (2.88, 4.33)]. Figure 4B shows that the associations between the classes of alcohol consumption and criminal behaviour at age 15 years and alcohol‐related problems only weaken very slightly with age. All results were very similar when adjusting for potential confounders (see Appendix S9). There was no evidence for an association between childhood CPs or classes of alcohol consumption and criminal behaviour with the linear slope or quadratic factor for alcohol‐related problems (results available on request), that is childhood and adolescent behaviours did not predict the rate of change of alcohol‐related problems in emerging adulthood.

#### Indirect effects of conduct problem latent classes (X) on alcohol‐related problems across emerging adulthood (Y) via the latent classes of alcohol consumption and criminal behaviour (M)

There was strong evidence for an indirect effect of ‘early‐onset persistent’ CPs on alcohol‐related problems at age 18 years via the latent classes of alcohol consumption and criminal behaviour in adolescence [*b*(95% CI) = 1.12 (0.52, 1.72)] and no evidence for a remaining direct effect [*b*(95% CI) = 0.04 (−0.85, 0.92)]. In contrast to this, there was little evidence for an indirect effect of ‘adolescent‐onset’ CPs on alcohol‐related problems at age 18 years via the latent classes of alcohol consumption and criminal behaviour in adolescence [*b*(95% CI) = 0.35 (−0.36, 1.07)] and little evidence for a remaining direct effect [*b*(95% CI) = 0.96 (−0.26, 2.18)]. Total, direct and indirect effects are shown in Appendix S10. Appendix S10 also shows that conclusions from unweighted analyses using bootstrapping were the same.

## Discussion

In this UK population‐based sample, youth who had ‘early‐onset persistent’ and ‘adolescent‐onset’ CPs showed higher levels of alcohol‐related problems at age 18 years compared to those with ‘low’ CPs. However, the associations weakened substantially over time, with little evidence of a total effect on alcohol‐related problems after age 19 years. ‘Early‐onset persistent’ CPs were also strongly associated with classes of alcohol consumption and criminal behaviour at age 15 years. Specifically, this group had over a threefold increased odds of reporting co‐occurring alcohol consumption and criminal behaviour, and of criminal behaviour without alcohol consumption compared to reporting neither. Additionally, youth who reported heavy alcohol consumption or criminal behaviour at age 15 years had higher levels of alcohol‐related problems at all ages across emerging adulthood compared with those who reported neither. Finally, there was a strong indirect effect of ‘early‐onset persistent’ CPs on alcohol‐related problems across emerging adulthood via the latent classes of age 15 alcohol consumption and criminal behaviour. By contrast, there was weaker evidence for an indirect effect of ‘adolescent‐onset’ CPs on alcohol‐related problems in emerging adulthood via alcohol consumption and/ or criminal behaviour in adolescence.

The main strengths of this study include the use of a large, prospective sample with data available for both males and females across childhood, adolescence and emerging adulthood. This allowed us to take a developmental perspective and identify antecedents of harmful levels of drinking in adulthood from various prior stages of the life course, both before and alongside the initiation of alcohol use. However, the findings need to be considered in the context of several limitations. First, as with most cohort studies, there was selective attrition over time. Proportionally few cohort members provided data on all measures across childhood, adolescence and emerging adulthood and both the exposure and confounders predicted missingness. This would have resulted in the most problematic cases (for example, those with childhood CPs) being excluded from the analyses and this may have impacted on the findings. Analyses were performed using FIML estimation, which allowed approximately 3,000 participants to be included, and IPW was used to address bias due to those lost from the analysis sample. However, these methods cannot always fully address any bias present from selective attrition. Additionally, a lack of power may still have impacted on our ability to detect associations that were smaller in magnitude, such as the indirect effect for the ‘adolescent‐onset’ group. Second, we focused specifically on co‐occurring heavy alcohol use and criminal behaviour at age 15 years as the mediator. It is possible that stronger associations with childhood CPs would have been observed had we assessed these behaviours earlier in adolescence, or examined chronicity across adolescence (Heron et al., 2013); however, we wanted to ensure correct temporal ordering between the exposure and mediator and focus on an age when both heavy alcohol use and criminal behaviour were assessed simultaneously. Additionally, there are other potential mediators (such as poorer educational attainment, unemployment, peer and family relationships, and depression) not examined here which may also contribute to explaining the associations observed, especially for the adolescent‐onset group. Third, although results were similar when adjusting for key confounders (including child sex, socio‐demographic factors and adverse childhood experiences), we cannot rule out the possibility of residual confounding due to measurement error or unmeasured confounders. Fourth, ALSPAC participants were recruited from the South‐West of England, and therefore, results do not necessarily generalise to the rest of the United Kingdom. Finally, those with early‐onset persistent CPs who also engaged in crime and/or heavy alcohol use in adolescence constitutes only 4.4% of the sample. Given the small size of this group within the current sample, there may be chance differences from those with early‐onset CPs who engage in crime and/or heavy alcohol use in the target population which could affect the accuracy of the results.

The findings from the present study support a recent systematic review and metaanalysis showing an association of similar strength for ‘early‐onset persistent’ and ‘adolescent‐onset’ CPs with excessive alcohol consumption in emerging adulthood, compared to ‘low’ CPs (Bevilacqua et al., 2017). Previous studies that have examined the association between developmental trajectories of CPs and alcohol‐related problems in adulthood have mainly focused on binary measures of binge drinking (Bor et al., 2010), harmful use (Kretschmer et al., 2014), and dependence or problems (Moffitt et al., 2002; Odgers et al., 2008) measured at a single point in time. The present study contributes to this literature by showing that the total effect for both ‘early‐onset persistent’ and ‘adolescent‐onset’ CPs on alcohol‐related problems weakens substantially across emerging adulthood, with little association after age 19 years (when alcohol‐related problems are increasing). This finding was unexpected, especially for those with ‘early‐onset persistent’ CPs, and it may be that excessive drinking in the early 20s is explained by more proximal factors, such as concurrent mental health problems, or significant life events; alternatively, it may be because excessive drinking becomes normative at this age in the United Kingdom, meaning that risk factor effects are diluted. Additionally, the weakening of associations with problematic alcohol use could also be explained by transitions to college or university given that the early 20s is when drinking patterns diverge most for those that do and do not attend university (Derefinko et al., 2016; O’Malley & Johnston, 2002; Reckdenwald et al., 2016) and therefore drinking assessments at this age are thought to be more unstable than those occurring slightly earlier or later making prediction more difficult (Zucker, 2008).

Those that reported heavy alcohol consumption and/or criminal behaviour at age 15 years had higher levels of alcohol‐related problems at all ages across emerging adulthood compared with those that reported neither. These findings support previous studies highlighting the importance of early‐onset alcohol consumption in the development of alcohol‐related problems in adulthood (Buchmann et al., 2009; Englund et al., 2008; Grant & Dawson, 1997; Hingson et al., 2006; Pitkänen et al., 2005). For example, two US national surveys found that those who initiated alcohol use in adolescence were more likely to experience alcohol abuse and dependence than those who initiated alcohol use in adulthood (Grant & Dawson, 1997; Hingson et al., 2006). These findings could reflect a potentially causal association with early‐onset alcohol use affecting developmental processes or alterations in brain function and structure which then leads to heavier and more frequent drinking in emerging adulthood. Alternatively, early alcohol consumption could be a marker of risk for later alcohol‐related problems, perhaps reflecting underlying behavioural dysregulation and under control (Buchmann et al., 2009; Englund et al., 2008; King & Chassin, 2007; Zucker, 2008). These potential explanations are not mutually exclusive, and both could be important, even within an individual. Interestingly, those who reported criminal behaviour without co‐occurring alcohol consumption had similar levels of alcohol‐related problems across emerging adulthood to those who reported heavy alcohol consumption in adolescence. This group, comprising 20% of the sample, may be an important group to investigate in future research given their high levels of alcohol‐related problems across emerging adulthood despite low risk for drinking heavily in adolescence.

As expected, most of the association between ‘early‐onset persistent’ CPs and alcohol‐related problems across emerging adulthood was explained through a combination of heavy alcohol consumption and/ or criminal behaviour at age 15 years. There was a strong indirect effect of ‘early‐onset persistent’ CPs on alcohol‐related problems at age 18 years, which explained 97% of the total effect, and this effect only weakened very slightly with age. However, a smaller proportion of the association between ‘adolescent‐onset’ CPs and later alcohol‐related problems was explained through these problem behaviours in adolescence (with the indirect effect explaining 27% of the total effect of ‘adolescent‐onset’ CPs on alcohol‐related problems at age 18 years). This was surprising given both heavy alcohol use and gaining a criminal record are thought to act as potential ‘snares’, trapping those with ‘adolescent‐onset’ CPs into experiencing persisting problems into adulthood (Moffitt, 1993). It may be that those with ‘adolescent‐onset’ CPs are more likely to engage in minor forms of problem behaviour that are more normative and age appropriate (Reckdenwald et al., 2016). For example, age 18 years is a time when most young people are drinking heavily, so the association between ‘adolescent‐onset’ CPs and alcohol‐related problems could reflect ongoing peer group influences.

## Conclusions

Alcohol‐related problems in emerging adulthood may be the consequence of a long‐term developmental process with early‐onset and persistent CPs setting a pathway to criminal behaviour and alcohol consumption in adolescence, which then escalates to heavier and more frequent drinking. Given that associations for heavy alcohol consumption and criminal behaviour in adolescence with later alcohol‐related problems were much stronger and stable across emerging adulthood, compared with those for early CPs, these problem behaviours in adolescence might represent important targets for prevention. As expected, the presence of co‐occurring heavy alcohol use and criminal behaviour showed the strongest association with later alcohol‐related problems suggesting that multicomponent prevention programmes focusing on these problem behaviours in adolescence might be beneficial. However, early intervention strategies for childhood CPs (for example, focusing on improving social and emotional development in the child and targeting parent factors) are also important to consider given that CPs were a precursor to both adolescent problem behaviours and later alcohol‐related problems.

## Supporting information


**Appendix S1.** Assessment of potential confounders and associations with alcohol‐related problems across emerging adulthood.
**Appendix S2** **. **Detail on the derivation of the mediator using latent class analysis.
**Appendix S3** **. **Detail on the counterfactual approach to mediation.
**Appendix S4** **. **Flow chart of retention in ALSPAC.
**Appendix S5** **. **Detail on inverse probability weighting (IPW) used to address missing data.
**Appendix S6** **. **Descriptive data on observed variables used in analyses to derive exposure, mediator and outcome.
**Appendix S7** **. **Detail on the quadratic latent growth curve for alcohol‐related problems.
**Appendix S8** **. **Overlap between the exposure latent classes (developmental trajectories of childhood conduct problems; CPs) and mediator latent classes (heavy alcohol consumption and criminal behaviour in adolescence); *N* = 3,038.
**Appendix S9** **. **Results adjusted for potential confounders.
**Appendix S10** **. **Total, direct and indirect effects of childhood conduct problems (CPs) on alcohol‐related problems across emerging adulthood.
