# Supplementary material for: Externalising pathways to alcohol‐related problems in emerging adulthood
Source: J Child Psychol Psychiatry. 2019 Nov 25;61(6):721–31. doi: 10.1111/jcpp.13167 (PMC7242151; doi:10.1111/jcpp.13167)
Supplement: Supplementary file 1 — Appendix S1. Assessment of potential confounders and associations with alcohol‐related problems across emerging adulthood. Appendix S2 . Detail on the derivation of the mediator using latent class analysis. Appendix S3 . Detail on the counterfactual approach to mediation. Appendix S4 . Flow chart of retention in ALSPAC. Appendix S5 . Detail on inverse probability weighting (IPW) used to address missing data. Appendix S6 . Descriptive data on observed variables used in analyses to derive exposure, mediator and outcome. Appendix S7 . Detail on the quadratic latent growth curve for alcohol‐related problems. Appendix S8 . Overlap between the exposure latent classes (developmental trajectories of childhood conduct problems; CPs) and mediator latent classes (heavy alcohol consumption and criminal behaviour in adolescence); N = 3,038. Appendix S9 . Results adjusted for potential confounders. Appendix S10 . Total, direct and indirect effects of childhood conduct problems (CPs) on alcohol‐related problems across emerging adulthood. [file JCPP-61-721-s001.docx]

**Supporting information – Externalising pathways to alcohol-related problems in emerging adulthood – by Hammerton *et al*.**

**Appendix S1.** Assessment of potential confounders and associations with alcohol-related problems across emerging adulthood.

*Assessment of potential confounders*

Maternal questionnaires completed during pregnancy or in the early postnatal period were used to assess maternal level of education (no high school qualifications; high school only; beyond high school) and parity (study child 1^st^, 2^nd^, 3^rd^ or subsequent child born in family). Adverse childhood experiences (ACEs) were assessed using parental questionnaires completed between child birth and child age 4 years including multiple questions on ‘classic’ ACEs (child sexual, physical or emotional abuse, substance abuse by the parents, parental mental illness or suicide attempt, violence between parents, parental separation, and parental criminal conviction) and two additional ACEs (satisfaction with neighbourhood and financial difficulties). These questions were used to derive a binary construct for exposure to each ACE and these were then summed to give a cumulative measure of childhood adversity (range 0 to 8). For further detail on the derivation of ACEs see Houtepen et al, 2018.

*Associations between potential confounders and alcohol-related problems*

Multivariable associations between child sex, sociodemographic measures, and ACEs and alcohol-related problems growth factors (the intercept, linear slope and quadratic factor) are shown in Appendix S1 Table 1 below. Associations between a covariate and the rate of change for an outcome can be difficult to interpret in the context of a quadratic latent growth curve. Therefore, associations are also shown between the confounders and the latent intercept fixed at age 20 and at age 22 years (representing average levels of alcohol-related problems at these ages).

As can be seen in Appendix S1 Table 1, when a confounder (e.g. maternal education) has a positive association with the intercept at age 18 years and the linear slope and a negative association with the quadratic factor, this translates to an effect that increases (becomes stronger) over time and then decreases again (e.g. high maternal education has a larger positive association with alcohol-related problems at age 20 compared to age 18 or 22 years). In contrast to this, when a confounder (e.g. sex) has a negative association with the intercept at age 18 years and the linear slope and no association with the quadratic factor, this translates to an effect that decreases (but again becomes stronger) over time (e.g. female sex has a larger negative association with alcohol-related problems at age 22 years compared to age 20 or 18 years). Finally, when there is no association between a confounder and the linear slope or quadratic factor (e.g. parity), this translates to an effect that remains constant over time (e.g. parity has a similar association with alcohol-related problems at age 18, 20 and 22 years).

Given the goal of this study to examine whether associations with alcohol-related problems persist or weaken across emerging adulthood, and the complexity involved in interpreting associations with the linear slope and quadratic factor, all further analyses focus on associations between an exposure and the latent intercept fixed at each age of the latent growth curve.

**Appendix S1 Table 1.** Multivariable associations between potential confounders and alcohol-related problems growth factors (intercept, linear slope, and quadratic); *N* = 3,038

|  | Growth factors for alcohol-related problems; unstandardised coefficient (95% confidence interval) | | | | |
| --- | --- | --- | --- | --- | --- |
| **Sociodemographic** | Intercept age 18 | Linear slope | Quadratic | Intercept age 20 | Intercept age 22 |
| Sex |  |  |  |  |  |
| Female | -0.33 (-0.70, 0.04) | -0.25 (-0.55, 0.05) | 0.00 (-0.06, 0.07) | -0.82 (-1.27, -0.36) | -1.28 (-1.71, -0.84) |
|  | p = 0.077 | p = 0.107 | p = 0.918 | p < 0.001 | p < 0.001 |
| Maternal education |  |  |  |  |  |
| High school | 0.94 (0.38, 1.51) | 0.42 (-0.04, 0.89) | -0.12 (-0.21, -0.02) | 1.33 (0.67, 1.99) | 0.80 (0.13, 1.46) |
| Beyond high school | 0.82 (0.30, 1.33) | 1.18 (0.74, 1.63) | -0.23 (-0.32, -0.13) | 2.28 (1.65, 2.91) | 1.94 (1.32, 2.56) |
|  | p = 0.003 | p < 0.001 | p < 0.001 | p < 0.001 | p < 0.001 |
| Parity |  |  |  |  |  |
| 2^nd^ child | 0.14 (-0.27, 0.54) | -0.01 (-0.34, 0.32) | 0.00 (-0.07, 0.07) | 0.11 (-0.39, 0.60) | 0.08 (-0.39, 0.55) |
| 3^rd^ child or more | 0.02 (-0.52, 0.57) | -0.17 (-0.60, 0.27) | 0.04 (-0.05, 0.12) | -0.17 (-0.82, 0.48) | -0.07 (-0.71, 0.56) |
|  | p = 0.797 | p = 0.739 | p = 0.690 | p = 0.727 | p = 0.894 |
| **Adverse childhood experiences** |  |  |  |  |  |
| Cumulative score | -0.01 (-0.20, 0.18) | 0.09 (-0.05, 0.23) | -0.02 (-0.04, 0.01) | 0.11 (-0.11, 0.32) | 0.09 (-0.12, 0.31) |
|  | p = 0.916 | p = 0.212 | p = 0.242 | p = 0.341 | p = 0.409 |

**Appendix S2.** Detail on the derivation of the mediator using latent class analysis.

Latent class analysis (LCA) was used to identify different patterns of alcohol consumption and criminal behaviour at age 15 years based on five binary measures (‘violent crime’, ‘non-violent crime’, ‘heavy typical drinking’, ‘frequent drinking’, and ‘regular binge drinking’). LCA assumes that variability in response is due to a latent (unobserved) grouping. Starting with a single class, a series of models were fitted, and theoretical and statistical steps were taken to decide on the optimal number of latent classes. Fit statistics included (a) the sample-size adjusted Bayesian information criterion (aBIC; Schwarz, 1978), (b) the Bootstrap Likelihood Ratio Test (BLRT; Nylund, Asparouhov, & Muthén , 2007) and the Lo, Mendell & Rubin Likelihood Ratio Test (LMR-LRT) (Lo, Mendell, & Rubin, 2001) which assesses the improvement in model fit for each additional class, and (c) bivariate model fit information—a test of the conditional independence assumption—using Pearson's χ^2^. Model fit statistics for the 1-class to the 5-class model are shown in Appendix S2 Table 1.

**Appendix S2 Table 1**. Fit statistics for latent classes of heavy alcohol consumption and criminal behaviour in adolescence; *N* = 5,133

| Classes | Parameters | Likelihood | aBIC | Entropy | Bivariate fit | BLRT | LMR-LRT |
| --- | --- | --- | --- | --- | --- | --- | --- |
| 1 | 5 | -12065 | 24157 | n/a | 4086 | *p* < 0.001 | *p* < 0.001 |
| 2 | 11 | -10858 | 21775 | 0.776 | 334 | *p* < 0.001 | *p* < 0.001 |
| 3 | 17 | -10664 | 21419 | 0.742 | 20 | *p* < 0.001 | *p* < 0.001 |
| **4** | **23** | **-10618** | **21359** | **0.712** | **1** | ***p* < 0.001** | ***p* < 0.001** |
| 5 | 29 | -10613 | 21382 | 0.792 | 0 | *p* = 0.150 | *p* = 0.188 |

aBIC: sample-size adjusted Bayesian information criterion (lower values indicate preferred models); BLRT: Bootstrap Likelihood Ratio Test; LMR-LRT: Lo, Mendell & Rubin Likelihood Ratio Test (high values indicate no evidence of improvement in fit from model with one less class)

The four-class model provided the best fit to the data. A range of socio-demographic, parental and child factors hypothesised to be closely associated with heavy alcohol consumption, criminal behaviour or both in adolescence were used as validation criteria. Maternal questionnaires completed during pregnancy were used to assess maternal level of education (no high school qualifications; high school only; beyond high school). Parental crime and problematic alcohol use were measured on eight occasions from the child’s birth to 11 years with questionnaires sent to mothers and their partners asking whether either had occurred since the last assessment. Any positive endorsement from either parent of being in trouble with the law/convicted was coded as positive for parental crime and any report of alcoholism/alcohol problems across the same time period was coded as positive for parental problematic alcohol use. Finally, during the computer-based session at a focus clinic at age ~15 years, respondents reported whether they had used cannabis in the last 12 months, and whether they currently smoked cigarettes weekly.

Multivariable associations between these validation criteria and the latent classes of alcohol consumption and criminal behaviour are shown in Appendix S2 Table 2 below.

**Appendix S2 Table 2.** Multivariable associations between sociodemographic measures (in pregnancy), parental crime and problematic alcohol use (from birth to age 11 years), cannabis and cigarette use (at age 15 years) and latent classes of heavy alcohol consumption and criminal behaviour; showing multinomial odds ratio (95% confidence interval); *N* = 4,767

|  | Latent classes of heavy alcohol consumption and criminal behaviour | | | |  |
| --- | --- | --- | --- | --- | --- |
| **Sociodemographic factors** | Neither | Crime only | Alcohol only | Alcohol and crime | *p value* |
| Sex |  |  |  |  |  |
| Female (53%) | Reference | 0.44 (0.34, 0.57) | 1.89 (1.19, 2.99) | 0.54 (0.25, 0.50) | < 0.001 |
| Maternal education |  |  |  |  |  |
| High school (35%) | Reference | 0.83 (0.59, 1.18) | 0.77 (0.44, 1.35) | 0.91 (0.58, 1.45) | 0.013 |
| Beyond high school (46%) | Reference | 0.74 (0.53, 1.03) | 1.06 (0.63, 1.77) | 0.53 (0.34, 0.83) |  |
| **Parental factors** |  |  |  |  |  |
| Parental crime |  |  |  |  |  |
| Yes (13%) | Reference | 1.29 (0.92, 1.80) | 0.96 (0.54, 1.73) | 1.64 (1.04, 2.59) | 0.140 |
| Parental alcohol use |  |  |  |  |  |
| Yes (8%) | Reference | 1.71 (1.12, 2.62) | 1.14 (0.58, 2.24) | 1.18 (0.68, 2.04) | 0.085 |
| **Child factors** |  |  |  |  |  |
| Cannabis use in last year |  |  |  |  |  |
| Yes (20%) | Reference | 10.71 (7.12, 16.10) | 10.31 (5.95, 17.85) | 53.68 (35.01, 82.20) | < 0.001 |
| Weekly cigarette use |  |  |  |  |  |
| Yes (10%) | Reference | 18.69 (7.98, 43.76) | 16.56 (6.26, 43.78) | 39.92 (17.02, 93.65) | < 0.001 |

**Appendix S3.** Detail on the counterfactual approach to mediation.

The counterfactual approach is based on conceptualising ‘potential outcomes’ for each individual [Y(*x*)] that would have been observed if particular conditions were met (i.e. had the exposure X been set to the value *x* through some intervention) – regardless of the conditions that were in fact met for each individual (VanderWeele, 2015). Mediation effects were estimated in a structural equation modelling framework based on a linear and a multinomial logistic regression model involving outcome *y* (the latent intercept for alcohol-related problems), exposure *x* (latent classes of childhood conduct problems), and mediator *m* (latent classes of heavy alcohol consumption and criminal behaviour):

E(*Y* | *x, m*)} = *θ*_0_ + *θ*_1_*x* + *θ*_2_*m*

logit {P*(M* = 1 | *x*)} = *β*_0_ + *β*_1_*x*

Specifically, we estimated the natural direct effect (NDE) and natural indirect effect (NIE). The method used to derive these effects with a nominal mediator has been described in detail elsewhere (Muthén, 2011); briefly, the estimated parameters from the linear regression model for Y and the multinomial logistic regression model for M were used to derive the expected value of the outcome given the exposure and mediator, i.e. E(*Y* | *X* = *x*, *M* = *m*), and the probability of the mediator being present given the exposure, i.e. P(*M* = 1 | *X* = *x*). Potential outcomes, E[*Y*(*x*, *M*(*x'*))] were then derived and the unstandardised beta coefficients for the NDE and NIE were calculated using direct application of the mediation formula (Pearl, 2012):

E[*Y*(0, *M*(0))] = E(*Y* | *X* = 0, *M* = 0)*[P(*M* = 0| *X* = 0)] + E(*Y* | *X* = 0, *M* = 1)*[P(*M* = 1 |*X* = 0)] + E(*Y* | *X* = 0, *M* = 2)*[P(*M* = 2| *X* = 0)] + E(*Y* | *X* = 0, *M* = 3)*[P(*M* = 3| *X* = 0)]

E[*Y*(1, *M*(0))] = E(*Y* | *X* = 1, *M* = 0)*[P(*M* = 0| *X* = 0)] + E(*Y* | *X* = 1, *M* = 1)*[P(*M* = 1| *X* = 0)] + E(*Y* | *X* = 1, *M* = 2)*[P(*M* = 2| *X* = 0)] + E(*Y* | *X* = 1, *M* = 3)*[P(*M* = 3| *X* = 0)]

E[*Y*(1, *M*(1))] = E(*Y* | *X* = 1, *M* = 0)*[P(*M* = 0| *X* = 1)] + E(*Y* | *X* = 1, *M* = 1)*[P(*M* = 1| *X* = 1)] + E(*Y* | *X* = 1, *M* = 2)*[P(*M* = 2| *X* = 1)] + E(*Y* | *X* = 1, *M* = 3)*[P(*M* = 3| *X* = 1)]

The NDE is the direct (unmediated) effect of the exposure on the outcome when the mediator takes the value it would take in the absence of the exposure. It is thus modelled as the direct effect of exposure *X* = 1 (e.g. ‘early-onset persistent’ conduct problems) versus the absence of exposure *X* = 0 (e.g. ‘low or childhood-limited’ conduct problems) on outcome *Y* (e.g. the latent intercept for alcohol-related problems) if mediator *m* (e.g. latent classes of alcohol consumption and criminal behaviour) were set to whatever it would be for *X* = 0.

NDE = E[*Y*(1, *M*(0))] - E[*Y*(0, *M*(0))]

The NIE captures the effect of the exposure on the outcome that operates by changing the mediator. It is thus modelled as the effect on outcome *Y* if the exposure were fixed at *X* = 1 and mediator *m* were changed from the level it would take if *X* = 0 to the level it would take if *X* = 1.

NIE = E[*Y*(1, *M*(1))] - E[*Y*(1, *M*(0))]

**Appendix S4.** Flowchart of retention in ALSPAC.

Initial sample after exclusions

*N* = 13,793

Data available for at least 1 of 4 repeated measures of alcohol-related problems

*N* = 6,178

Pregnant women enrolled in ALSPAC Phase 1

*N* = 14,541

Complete data on confounders, exposure and mediator

*N* = 3,038

Exclusion criteria: multiple births

Sample used in main analyses

Invited to take part in at least 1 of 4 repeated assessments for alcohol-related problems

*N* = 10,601

Maximum sample used to derive weights

Offspring alive at one year

*N* = 13,988

**Appendix S5.** Detail on inverse probability weighting (IPW) used to address missing data

IPW has been recommended over alternative methods for dealing with missing data (such as multiple imputation) in situations where whole blocks of data are missing for a large proportion of individuals (Seaman et al, 2012). A first set of weights were derived from a logistic regression analysis between a set of measures assessed in pregnancy that were independently predictive of missing data and/ or variables in the analysis (maternal smoking in pregnancy, maternal education, housing tenure, maternal history of severe depression and child sex) and inclusion in the final sample (*N* = 3,038/ 10,601). Minimal missing data on indicators used to derive weights were singly imputed as the modal or mean value (all indicators had < 8% of values missing). The Hosmer-Lemeshow test was used assess the fit of the missingness model, with results showing no indication of poor fit (Hosmer-Lemeshow χ^2^ (df) = 8.93 (9); *p* = 0.26). Weights ranged from 2 to 27. A second set of weights were derived from a logistic regression analysis between the exposure (conduct problem trajectories) and adverse childhood experiences and inclusion in the final sample (*N* = 3,038/ 5,713). The Hosmer-Lemeshow test showed no indication of poor fit (Hosmer-Lemeshow χ^2^ (df) = 3.63 (7); *p* = 0.60). Weights ranged from 2 to 4. These two sets of weights were then multiplied together to create one final weight to use in the analysis models. Due to the potential for extreme weighted values adversely influencing subsequent analyses, weights were trimmed to the 90^th^ percentile (final weights ranged from 4 to 13).

Associations between indicators used to derive the IPW and inclusion in the analysis sample are shown in Appendix S5 Table 1.

**Appendix S5 Table 1.** Associations between indicators used to derive the IPW and inclusion in the analysis sample; showing % (n) or mean (standard deviation) as appropriate and odds ratio (95% confidence interval) with outcome coded 0 “in analysis sample” and 1 “not in analysis sample”

|  | In analysis sample | Not in analysis sample | OR (95% CI) | p value |
| --- | --- | --- | --- | --- |
| **Sociodemographic and parental factors in pregnancy** | *N* = 3,038 | *N* = 7,563 |  |  |
| Child sex |  |  |  |  |
| Female | 56% (1,690) | 48% (3,615) | 0.73 (0.67, 0.79) | < .001 |
| Maternal education |  |  |  |  |
| No high school | 14% (436) | 29% (2,207) | Reference |  |
| High school | 34% (1,029) | 31% (2,377) | 0.46 (0.40, 0.52) | < .001 |
| Beyond high school | 52% (1,573) | 39% (2,979) | 0.37 (0.33, 0.42) |  |
| Maternal smoking |  |  |  |  |
| Yes | 13% (380) | 27% (2,006) | 2.52 (2.24, 2.84) | < .001 |
| Housing tenure |  |  |  |  |
| Mortgaged | 89% (2,703) | 74% (5,573) | Reference |  |
| Subsidised rented | 5% (154) | 16% (1,228) | 3.87 (3.25, 4.60) | < .001 |
| Private rented | 6% (181) | 10% (762) | 2.04 (1.73, 2.42) |  |
| Maternal severe depression |  |  |  |  |
| Yes | 5% (151) | 9% (655) | 1.81 (1.51, 2.18) | < .001 |
| **Conduct problems (exposure)** | *N* = 3,038 | *N* = 2,675 |  |  |
| Low | 75% (2,273) | 68% (1,808) | Reference |  |
| Childhood-limited | 11% (335) | 13% (335) | 1.26 (1.07, 1.48) | <.001 |
| Adolescent-onset | 7% (219) | 9% (251) | 1.44 (1.19, 1.74) |  |
| Early-onset persistent | 7% (211) | 11% (281) | 1.67 (1.39, 2.02) |  |
| **Adverse childhood experiences (confounder)** | *N* = 3,038 | *N* = 2,675 |  |  |
| Cumulative score | 0.73 (1.06) | 0.95 (1.25) | 1.18 (1.12, 1.23) | < .001 |

**Appendix S6.** Descriptive data on observed variables used in analyses to derive exposure, mediator and outcome.

|  | Data available | Total | Males | Females |
| --- | --- | --- | --- | --- |
| **Exposure^1^**  **(n = 7,201)** | % (n) | Prevalence (n) | Prevalence (n) | Prevalence (n) |
| CPs age 4 | 96% (6,902) | 29% (1,987) | 30% (1,051) | 27% (936) |
| CPs age 7 | 95% (6,855) | 24% (1,613) | 25% (857) | 22% (756) |
| CPs age 8 | 92% (6,621) | 20% (1,314) | 22% (729) | 18% (585) |
| CPs age 10 | 94% (6,803) | 17% (1,145) | 19% (639) | 15% (506) |
| CPs age 12 | 88% (6,358) | 15% (978) | 17% (529) | 14% (449) |
| CPs age 13 | 84% (6,042) | 16% (962) | 16% (498) | 15% (464) |
| **Mediator^2^**  **(n = 5,133)** | % (n) | Prevalence (n) | Prevalence (n) | Prevalence (n) |
| Heavy typical drinking | 93% (4,795) | 21% (1,009) | 19% (434) | 22% (575) |
| Frequent drinking | 97% (4,980) | 19% (957) | 20% (474) | 18% (483) |
| Regular binge drinking | 97% (4,962) | 10% (515) | 11% (245) | 10% (270) |
| Violent crime | 98% (5,040) | 23% (1,180) | 33% (791) | 15% (389) |
| Nonviolent crime | 98% (5,037) | 24% (1,199) | 28% (659) | 20% (540) |
| **Outcome^3^**  **(n = 6,178)** | % (n) | Mean (standard deviation) | Mean (standard deviation) | Mean (standard deviation) |
| APs age 18 | 63% (3866) | 6.98 (4.86) | 7.23 (4.95) | 6.79 (4.78) |
| APs age 19 | 50% (3072) | 7.81 (5.06) | 8.02 (5.09) | 7.69 (5.03) |
| APs age 21 | 62% (3828) | 8.68 (5.54) | 9.35 (5.75) | 8.24 (5.36) |
| APs age 23 | 59% (3620) | 7.13 (4.79) | 7.98 (5.27) | 6.66 (4.44) |

^1^exposure is developmental trajectories of conduct problems (CPs) from age 4 to 13 years; ^2^mediator is latent classes of heavy alcohol use and criminal behaviour at age 15 years; ^3^outcome is latent growth curve of alcohol-related problems (APs) from age 18 to 23 years

**Appendix S7.** Detail on the quadratic latent growth curve for alcohol-related problems.

A quadratic latent growth curve was estimated to capture non-linear change from age 18 to 23 years. Appendix S7 Figure 1 shows observed and estimated means (with 95% confidence intervals) for alcohol-related problems across emerging adulthood.

**Appendix S7 Figure 1.** Observed and estimated means (with 95% confidence intervals) for alcohol-related problems across emerging adulthood; diamonds represent observed means; *N* = 6,178

Means, variances and correlations for alcohol-related problems growth factors in the unconditional model are shown in Appendix S7 Table 1 below.

**Appendix S7 Table 1.** Means, variances and correlations for alcohol-related problems (APs) growth factors in the unconditional model; showing parameter estimate (standard error); *N* = 6,178

|  | 1. | 2. | 3. |
| --- | --- | --- | --- |
| 1. APs intercept (at age 18 years) | 1 |  |  |
| 2. Linear slope | 0.06 (0.06) | 1 |  |
| 3. Quadratic factor | -0.29 (0.09) | -0.96 (0.01) | 1 |
| Mean | 7.20 (0.07) | 1.45 (0.06) | -0.30 (0.01) |
| Variance | 17.24 (0.73) | 3.43 (0.49) | 0.08 (0.02) |

**Appendix S8.** Overlap between the exposure latent classes (developmental trajectories of childhood conduct problems; CPs) and mediator latent classes (heavy alcohol consumption and criminal behaviour in adolescence); *N* = 3,038.

|  | **Mediator latent classes** | | | |
| --- | --- | --- | --- | --- |
| **Exposure latent classes** | Neither (65%)^1^ | Crime only (20%) | Heavy alcohol only (6%) | Heavy alcohol and crime (9%) |
| Low CPs (64%) | 44% (1342) | 11% (329) | 4% (133) | 5% (147) |
| Childhood-limited CPs (15%) | 10% (314) | 3% (79) | 1% (27) | 2% (49) |
| Adolescent-onset CPs (12%) | 7% (206) | 3% (97) | 0.5% (16) | 1% (30) |
| Early-onset persistent CPs (9%) | 4% (113) | 3% (102) | 0.4% (11) | 1% (41) |

^1^class prevalences based on sample used in analysis (*N* = 3,038) so may differ slightly from prevalences when deriving classes using all available data (exposure: *N* = 7,201; mediator *N* = 5,133)

**Appendix S9.** Results adjusted for potential confounders.

In mediation analyses, four assumptions are made with respect to confounding. These include no unmeasured confounders for any of the paths and no measured or unmeasured confounder for the association between mediator and outcome which lies on the causal pathway from the exposure. In the current analyses, the same set of factors were assumed to confound all paths, and these were all assessed, or reported to occur either before or simultaneously to the assessment of the exposure. Potential confounders included child sex, parity, maternal education, and adverse childhood experiences (including child physical, sexual and emotional abuse, violence between parents, parental substance use, mental health problems or suicide attempt, parental criminal offence, parental separation, financial difficulties and satisfaction with the neighbourhood). A directed acyclic graph (DAG) showing hypothesised relationships is shown in Appendix S9 Figure 1.

**Appendix S9 Figure 1.** Directed acyclic graph showing hypothesised relationships between variables in analysis

Alcohol-related problems

Conduct problems

Heavy alcohol use and/ or criminal behaviour

Child sex

Parity

Maternal education

Adverse childhood experiences

After adjusting for these potential confounders, there was still evidence for an association between childhood conduct problems and initial levels of alcohol-related problems at age 18 years (Wald χ^2^ (3) = 16.45; *p* < .001). Specifically, those with ‘early-onset persistent’ conduct problems [b(95% CI) = 1.37 (0.38, 2.36)] and those with ‘adolescent-onset’ conduct problems [b(95% CI) = 1.50 (0.33, 2.66)] had higher levels of alcohol-related problems at age 18 years compared to those with ‘low’ conduct problems.

Strong evidence also remained for an association between childhood conduct problems and classes of alcohol consumption and criminal behaviour (Wald χ^2^ (9) = 25.18; *p* = .003). Specifically, those with ‘early-onset persistent’ conduct problems compared to those with ‘low’ conduct problems had increased odds of reporting both heavy alcohol consumption and criminal behaviour at age 15 years [OR (95% CI) = 2.97 (1.44, 6.12)], and of criminal behaviour without heavy alcohol consumption [OR (95% CI) = 3.44 (1.84, 6.43)] compared to reporting neither.

Finally, there was still strong evidence of an association between classes of alcohol consumption and criminal behaviour and initial levels of alcohol-related problems at age 18 years (Wald χ^2^ (3) = 297.63; *p* < .001). Specifically, those that reported either heavy alcohol consumption or criminal behaviour at age 15 years had higher levels of alcohol-related problems at age 18 years compared to those that reported neither [both alcohol and crime: b(95% CI) = 5.86 (4.91, 6.83); alcohol only: b(95% CI) = 4.59 (3.22, 5.96); crime only: b(95% CI) = 3.62 (2.90, 4.35)].

**Appendix S10.** Total, direct and indirect effects of childhood conduct problems (CPs) on alcohol-related problems across emerging adulthood.

Appendix S10 Table 1 shows total, direct and indirect effects of childhood conduct problems (CPs) on alcohol-related problems at age 18 years via latent classes of heavy alcohol consumption and criminal behaviour in adolescence.

**Appendix S10 Table 1.** Total, direct and indirect effects of childhood conduct problems (CPs) on alcohol-related problems at age 18 years via latent classes of heavy alcohol consumption and criminal behaviour in adolescence; showing unstandardised coefficient (95% confidence interval); *N* = 3,038

| **Latent classes** | Total effect | Indirect effect | Direct effect |
| --- | --- | --- | --- |
| Low CPs | Reference | Reference | Reference |
| Childhood-limited CPs | 0.01 (-0.85, 0.86) | 0.12 (-0.45, 0.69) | -0.11 (-1.00, 0.77) |
| Adolescent-onset CPs | 1.31 (0.17, 2.45) | 0.35 (-0.36, 1.07) | 0.96 (-0.26, 2.18) |
| Early-onset persistent CPs | 1.16 (0.17, 2.14) | 1.12 (0.52, 1.72) | 0.04 (-0.85, 0.92) |

Appendix S10 Figure 1 shows the indirect effect (95% confidence interval) for early-onset persistent and adolescent-onset conduct problem on alcohol-related problems from age 18 to 22 years. As can be seen from the figure, in contrast to the total effects, the indirect effects only weaken very slightly with age.

**Appendix S10 Figure 1.** Indirect effect (95% confidence interval) for each conduct problem class on alcohol-related problems from age 18 to 22 years; panel A shows ‘early-onset persistent’ vs ‘low’ conduct problems, panel B shows ‘adolescent-onset’ vs ‘low’ conduct problems


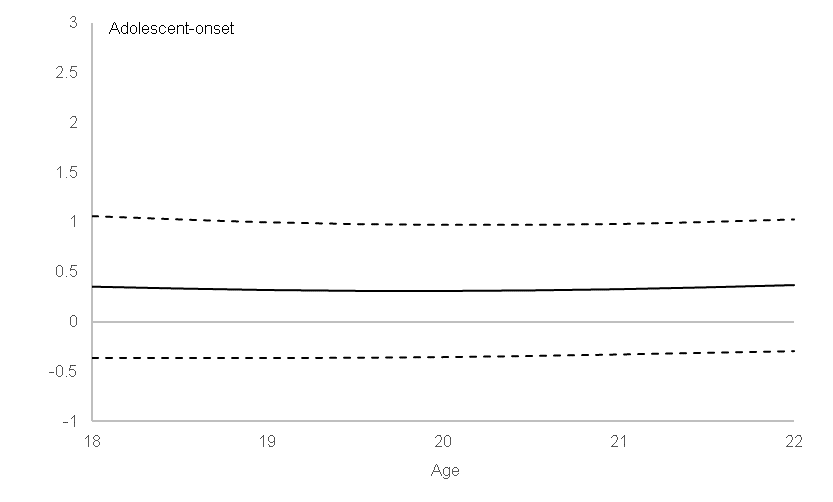

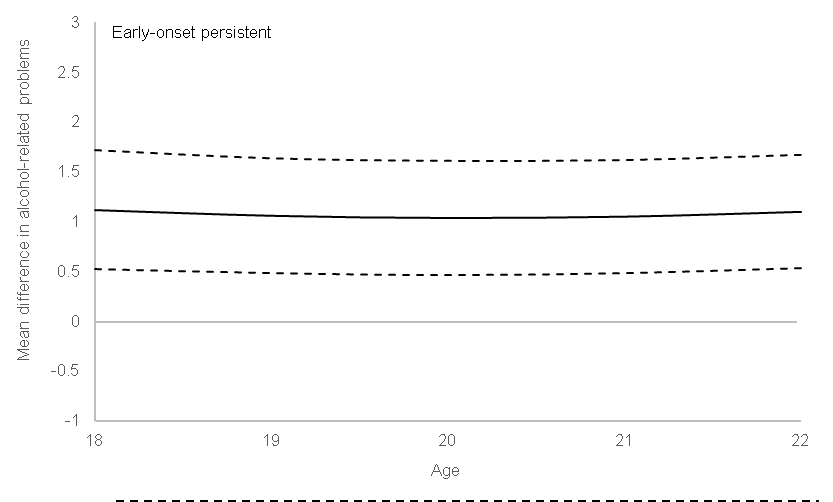


As it is not possible to incorporate bootstrapped confidence intervals and inverse probability weights into the same model, sensitivity analyses using percentile-based bootstrapped confidence intervals (with 2373 replications) are presented in Appendix S10 Table 2.

**Appendix S10 Table 2.** Unweighted total, direct and indirect effects of childhood conduct problems (CPs) on alcohol-related problems at age 18 years via latent classes of heavy alcohol consumption and criminal behaviour in adolescence; showing unstandardised coefficient (percentile-based bootstrap 95% confidence interval); *N* = 3,038

| **Latent classes** | Total effect | Indirect effect | Direct effect |
| --- | --- | --- | --- |
| Low CPs | Reference | Reference | Reference |
| Childhood-limited CPs | 0.22 (-0.35, 1.35) | 0.09 (-0.45, 0.71) | 0.13 (-0.53, 1.29) |
| Adolescent-onset CPs | 1.30 (0.35, 2.90) | 0.37 (-0.35, 1.19) | 0.93 (-0.24, 2.62) |
| Early-onset persistent CPs | 1.14 (0.45, 2.68) | 1.25 (0.67, 1.97) | -0.11 (-0.78, 1.25) |

**Supplementary references**

Houtepen, L.C., Heron, J., Suderman, M.J., Tilling, K. & Howe, L.D. (2018). Adverse childhood experiences in the children of the Avon Longitudinal Study of Parents and Children (ALSPAC). *Wellcome open research*, *3*, 106.

Lo, Y., Mendell, N.R., & Rubin, D.B. (2001). Testing the number of components in a normal mixture. *Biometrika,* *88*(3),767–778.

Muthén, B. (2011). Applications of causally defined direct and indirect effects in mediation analysis using SEM in Mplus (Technical Report). Los Angeles, CA: Muthén & Muthén

Nylund, K.L., Asparouhov, T., & Muthén, B.O. (2007). Deciding on the number of classes in latent class analysis and growth mixture modeling: A Monte Carlo simulation study. *Structural Equation Modeling,* *14*(4),535–569.

Pearl, J. (2012). The causal mediation formula—a guide to the assessment of pathways and mechanisms. *Prevention Science,* *13*(4),426-436.

Schwarz, G. (1978). Estimating the Dimension of a Model. *The Annals of Statistics, 6*(2),461-464.

Seaman, S.R., White, I.R., Copas, A.J., & Li, L. (2012). Combining Multiple Imputation and Inverse-Probability Weighting. *Biometrics*, 68, 129–137.

VanderWeele, T. (2015). *Explanation in causal inference: methods for mediation and interaction.* Oxford University Press.
